# Application of High-Frequency Ultrasound (48 MHz) in Imaging and Evaluating the Effectiveness of Microneedling in Reducing Striae Distensae—A Pilot Study

**DOI:** 10.3390/diagnostics16213444

**Published:** 2026-10-22

**Authors:** Marta Wacewicz-Muczyńska, Wiktoria Krzyżewska, Bogumiła Redlarska

**Affiliations:** 1 Department of Specialist Cosmetology, Faculty of Pharmacy with the Division of Medical Analytics, Medical University of Bialystok, 15-267 Bialystok, Poland; bogumila.redlarska@umb.edu.pl; 2 Student Research Group, Department of Specialist Cosmetology, Faculty of Pharmacy with the Division of Medical Analytics, Medical University of Bialystok, 15-267 Bialystok, Poland; wiktoria.krzyzewska@onet.pl

**Keywords:** high frequency ultrasound, striae distensae, microneedling, echogenicity

## Abstract

**Background:** Striae distensae (stretch marks) are atrophic skin lesions. Microneedling stimulates collagen remodeling, and high-frequency ultrasonography (HFUS) allows for the evaluation of these tissue changes. **Objective:** To assess the effectiveness of microneedling in reducing abdominal striae distensae using photographic and HFUS imaging. **Methods:** Ten women underwent three microneedling treatments at 6–8 week intervals. Skin structure was evaluated before and after the therapy using HFUS and photography. **Results:** Ultrasound imaging revealed pronounced structural tissue remodeling and a visible reduction in lesion visibility across the cohort. However, quantitative ultrasound shifts did not reach statistical significance in this pilot sample (*n* = 10), indicating that these findings represent observational trend data. In seven patients, a more homogeneous and ordered collagen structure was observed (increased medium echogenicity), while three showed the opposite trend. Visually, all subjects experienced shallower stretch marks, improved skin texture, evened color, and observational improvement. **Conclusions:** High-frequency ultrasonography suggests that microneedling is a safe and promising method for tissue remodeling and the clinical reduction of striae distensae.

## 1. Introduction

Striae distensae (SD) are a common dermatological concern that, while medically benign, often cause significant psychological distress [1,2]. SD affect up to 90% of adolescents and pregnant women, exhibiting sex-specific anatomical distributions [3,4]. The etiopathogenesis of SD is multifactorial and complex, with the precise underlying mechanisms remaining unknown [2,5].

**Figure 1:**
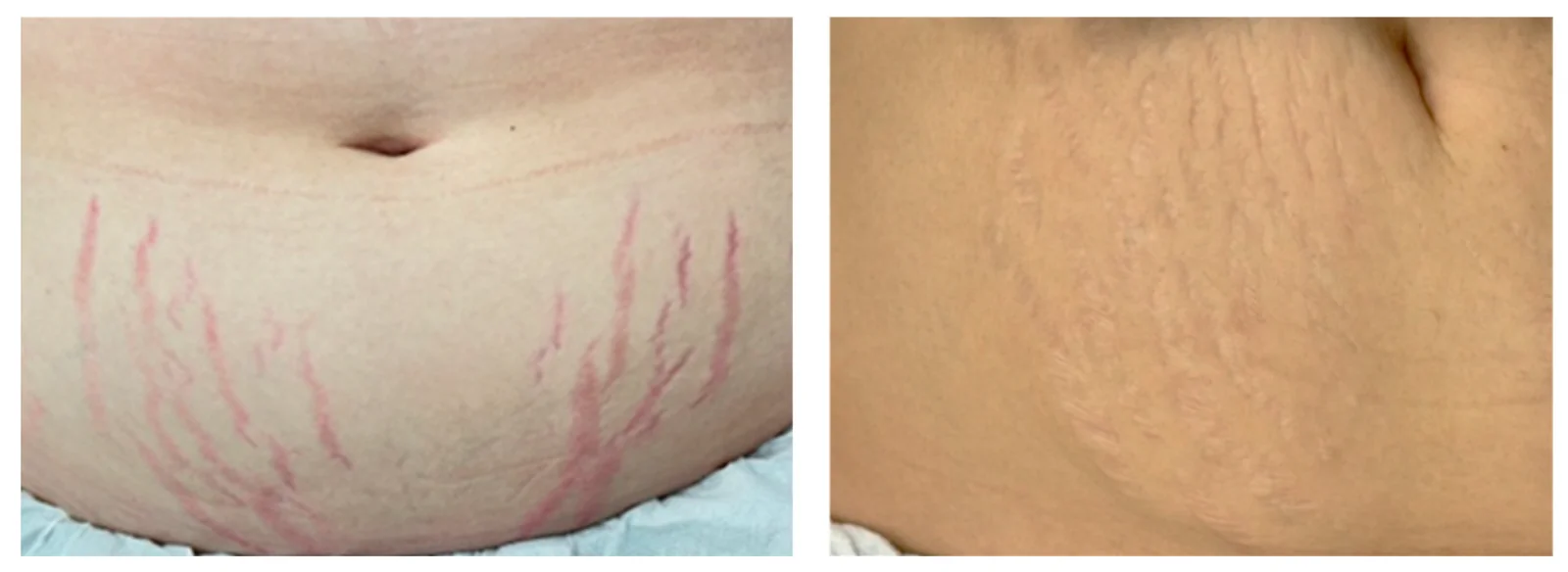
Striae rubrae (**left**) and striae albae (**right**) of patient.

Clinically and histologically, striae distensae exhibit a biphasic evolution. The initial active phase (striae rubrae) presents as erythematous lesions with perivascular inflammation (Figure 1) and realigned collagen [6,7,8,9]. Subsequently, lesions transition into a chronic phase (striae albae), characterized by scar-like hypopigmentation, epidermal atrophy, and severe disorganization of collagen and elastin networks [10,11,12].

Microneedling (percutaneous collagen induction) is a minimally invasive therapy that triggers wound-healing, promoting subepidermal neocollagenesis and elastogenesis [13,14,15]. Due to its documented efficacy in atrophic scar remodeling [16,17,18], it is hypothesized to similarly improve striae distensae. Nevertheless, the current literature exhibits substantial variability in protocols, reported outcomes, and evaluation methods, which range from subjective scales to objective imaging [17,19,20,21,22].

Ultrasonography serves as a safe, non-invasive, and non-ionizing imaging modality for visualizing internal anatomical structures and pathological lesions [23,24,25]. Ultrasound precisely measures epidermal and dermal thickness by analyzing acoustic echoes based on tissue density and elasticity [26]. The resulting images display hyperechoic (bright) as well as hypoechoic or anechoic (dark) structures [27,28]. For dermatological applications, lower-frequency transducers (7.5–15 MHz) evaluate structures deeper than 4 cm, including subcutaneous tissue, lymph nodes, and cysts [23,29,30]. Conversely, higher-frequency systems (50–100 MHz) provide superior resolution for evaluating superficial skin layers (0.15–4 mm) [13,26,31,32,33,34]. Nevertheless, current striae imaging literature remains limited, primarily relying on 20–22 MHz transducers for basic epidermal measurements [24,35,36,37]. To the best of our knowledge, this work represents the first investigation utilizing a 48 MHz high-frequency ultrasound system in this context. 

This study aimed to evaluate the efficacy of microneedling in treating abdominal SD through quantitative measurement of lesion echogenicity using a 48 MHz ultrasound transducer.

## 2. Materials and Methods

### 2.1. Patients

The study cohort comprised 11 women aged 23–45 years presenting with abdominal SD of various etiologies (Table 1). One participant withdrew from the study due to personal reasons, leaving 10 patients who successfully completed the protocol. Prior to enrollment, all individuals completed a consultation questionnaire to assess eligibility and provided written informed consent to participate in the study. Before initiating treatment, baseline photographic and ultrasonographic documentation of the affected areas was performed. The study protocol was approved by the Bioethics Committee of the Medical University of Bialystok (Approval No. APK.002.202.2025).

### 2.2. Inclusion and Exclusion Criteria

Participants completed a health questionnaire covering medical history, medications, allergies, and skincare routines. Exclusion criteria included age under 18, pregnancy, lactation, diabetes, keloid tendency, active skin diseases, ongoing chemo- or radiotherapy, recent aesthetic procedures, and use of systemic corticosteroids, anticoagulants, or topical retinoids. Following clinical evaluation to rule out contraindications, eligible individuals were briefed on the protocol, potential adverse effects, and post-procedure care.

### 2.3. Ultrasound Examination

Skin parameters were assessed using a Dramiński DermaView (Dramiński S.A., Szabruk, Poland) ultrasound system equipped with a 48 MHz transducer. Pre- and post-treatment ultrasound evaluations were performed using a 48 MHz system (Dramiński DermaView, Poland) in B-mode. Scans displayed three distinct layers: a superficial hyperechoic epidermal band, an intermediate dermis of lower echogenicity, and a deep hypoechoic subcutaneous layer [28,38].

In this study, the clinical appearance and echogenicity of abdominal striae distensae were evaluated. The structural density of each lesion was quantified based on the ratio of bright pixels to the total number of pixels (total pixels, TP) within a designated region of interest (ROI) on the ultrasound image. Pixel density was classified into three echogenicity ranges: low-echogenic pixels (LEP; darker pixels, range 0–49), medium-echogenic pixels (MEP; range 50–150), and high-echogenic pixels (HEP; brighter pixels, range 151–255). Specifically, MEP and HEP pixel distributions were analyzed to assess tissue echogenicity [26,39]. Before starting the treatment, for each patient, a single area point was selected.

To minimize operator-dependent variability, all ultrasound scans were performed by the same experienced operator using identical equipment settings, gain, and frequency parameters. Measurements were consistently taken from the same localized anatomical points marked prior to the treatment sessions.

### 2.4. Protocol of Microneedling

**Figure 2:**
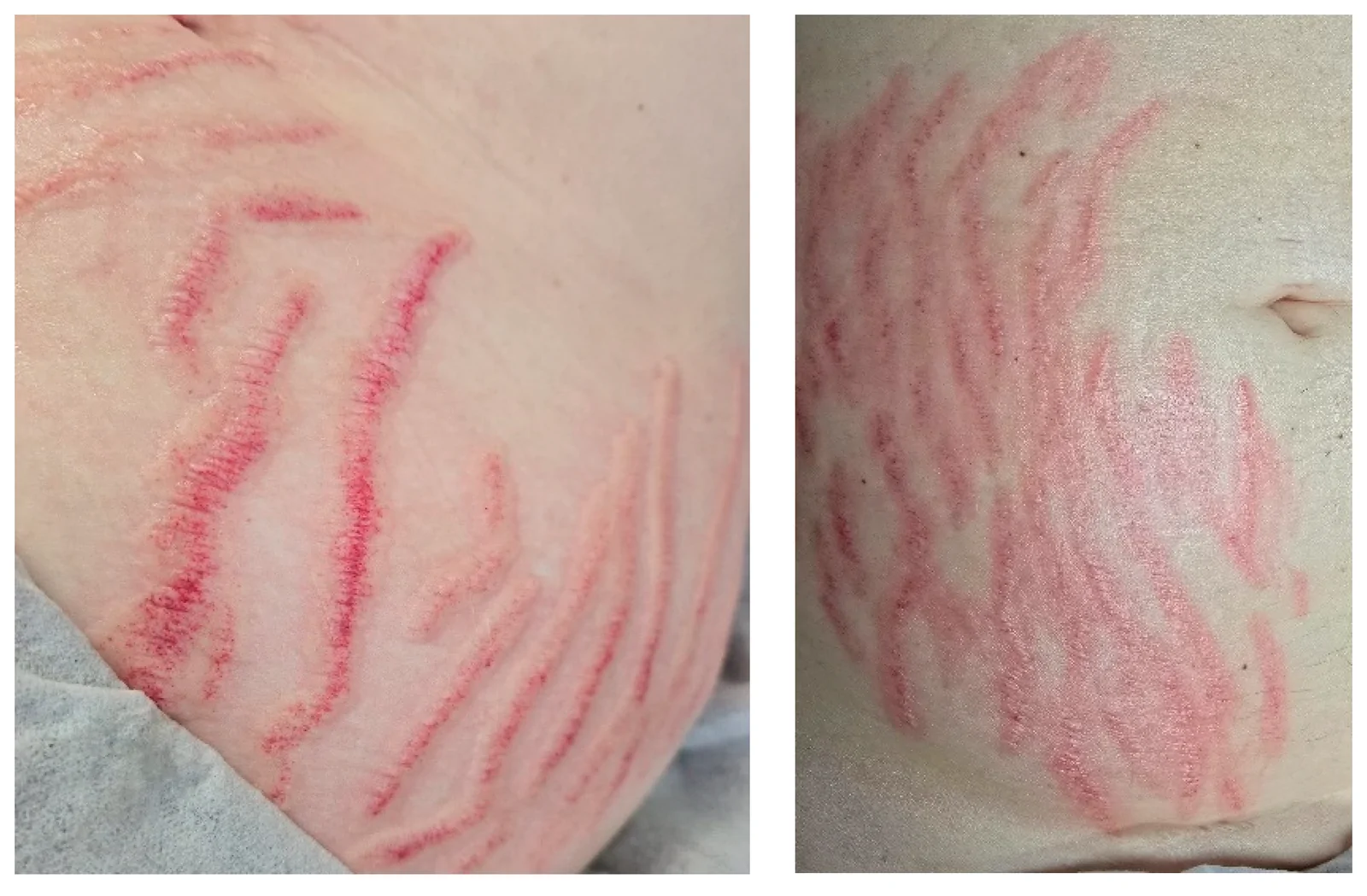
Steps of procedure-bleeding points were considered as the end of the treatment.

A total of three treatment sessions of microneedling were performed at 6–8-week intervals. The procedures were carried out using a Soft Liner Basic device designed for microneedling and micropigmentation, operating at a needle frequency of 90 to 150 punctures per second. Sterile, single-use needle cartridge systems designed for microneedling mesotherapy with a SEIRIN FLAT 7-type needles (SEIRIN Corporation, Shizuoka, Japan) (7 needles in a row, 0.30 mm thickness, 30 mm length) were used. Prior to the procedure, the target area was thoroughly cleansed and disinfected. Subsequently, microneedling was performed using perpendicular strokes across the striae (Figure 2). Treatments were performed using an auto microneedling device. The actual needle penetration depth into the skin was adjusted between 1.5 mm and 2.0 mm (depending on the anatomical site and baseline severity of the striae) to ensure optimal dermal stimulation. While the sterile needle cartridge component has a total manufactured physical length of 30 mm, the actual clinical skin penetration depth (1.5–2.0 mm) was precisely controlled and calibrated via the adjustable motor handpiece depth setting. Uniform erythema and pinpoint bleeding served as the primary clinical endpoints. Patients were instructed to use a moisturizer (Cicaplast, La Roche-Posay, Paris, France) two times daily for 2 weeks after treatment to promote wound healing and prevent dryness. HFUS evaluation was performed 8 weeks after the final session, matching our photographic follow-up interval. Photographs using identical camera settings, lighting, and positioning were taken before and 8 weeks after the last treatment.

### 2.5. Statistical Analyses

The results obtained were analyzed using the Statistica v.14.0. statistical program and Microsoft Office Excel 2019 software. Descriptive statistics for continuous variables were presented as median, and the first and third quartiles (Q1–Q3). Statistical analyses were performed using the Shapiro–Wilk test to assess the normality of the data. Due to the small sample size and non-normal distribution of the variables, non-parametric statistical methods were applied. The comparison of clinical parameters (MEP/TP, HEP/TP) between the pre-treatment and post-treatment phases was conducted using the Wilcoxon signed-rank test. A *p*-value of < 0.05 was considered statistically significant.

## 3. Results

### 3.1. Characteristics of Changes in Skin Echogenicity

Descriptive statistics for the study group’s clinical parameters before and after treatment are summarized in Table 2. For the MEP/TP parameter, the median increased from 72.52 pre-treatment to 81.25 post-treatment. Similarly, the median for the HEP/TP parameter shifted from 28.47 to 19.55 following therapy. Statistical analysis using the Wilcoxon signed-rank test revealed no statistically significant differences between pre- and post-treatment results for the MEP/TP (Z = 1.07, *p* = 0.285) and HEP/TP (Z = −0.85, *p* = 0.395) parameters.

**Figure 3:**
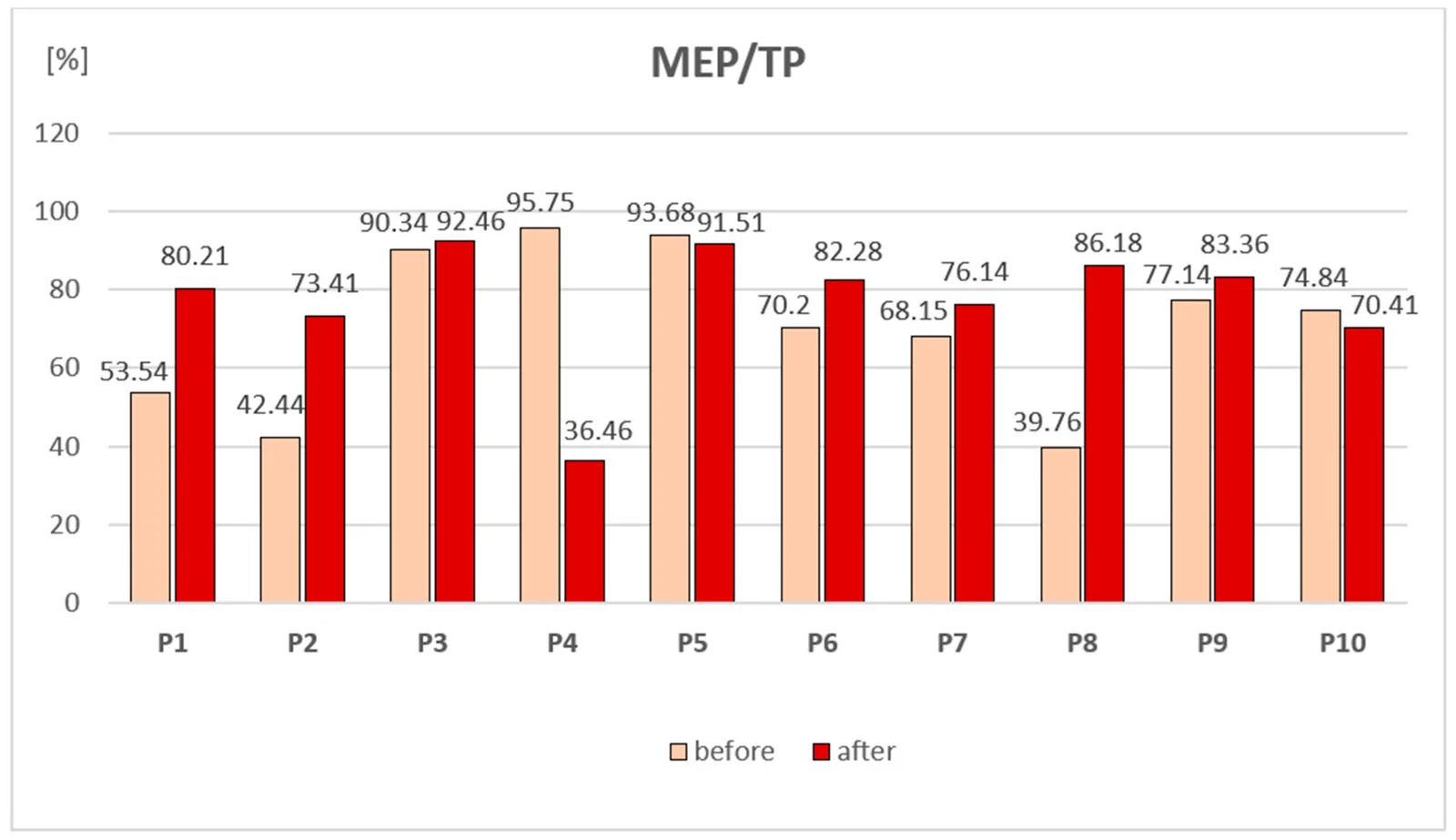
Comparison of the MEP/TP parameter values before and after therapy for individual patients.

**Figure 4:**
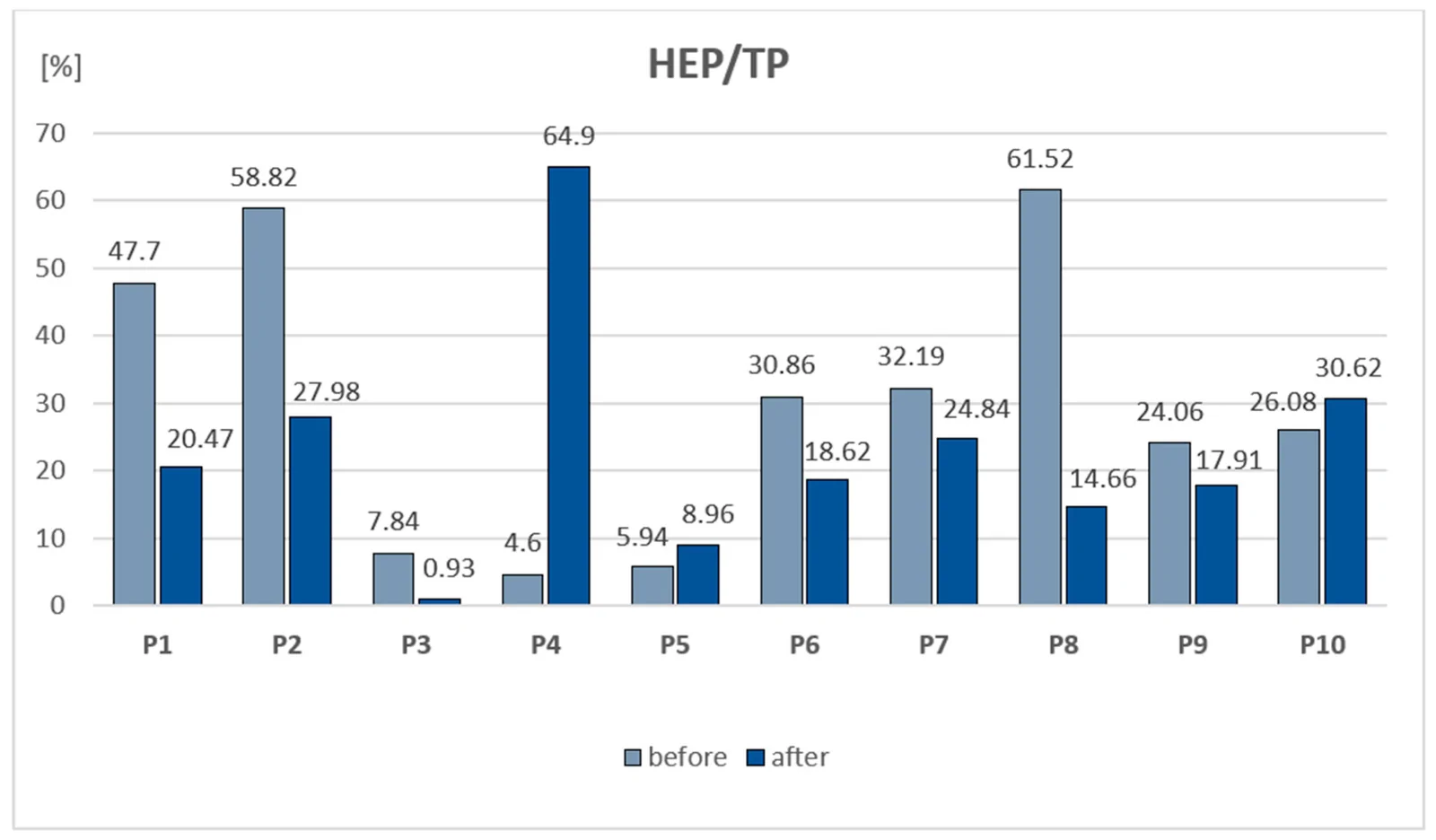
Comparison of the HEP/TP parameter values before and after therapy for individual patients.

As shown in Table 3, 7 of 10 subjects exhibited increased MEP/TP and decreased HEP/TP values, alongside individual variation. Figure 3 and Figure 4 illustrate individual pre- and post-therapy comparisons for the MEP/TP and HEP/TP parameters, respectively. Figure 3 displays the individual pre- and post-therapy results for the MEP/TP parameter. Figure 4 displays the individual pre- and post-therapy results for the HEP/TP parameter.

### 3.2. Visible and Ultrasound Changes in Striae Distensae

**Figure 5:**
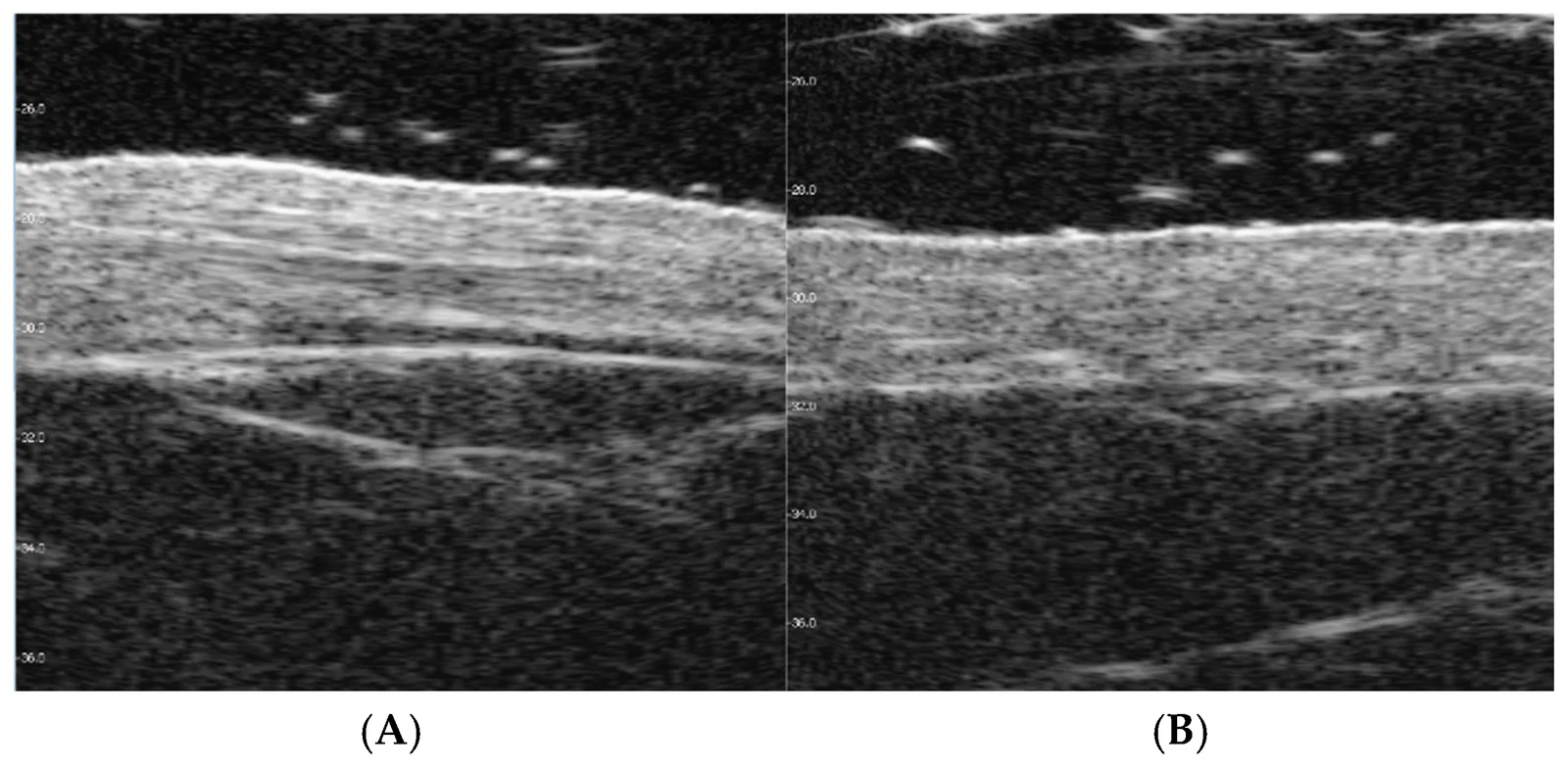
Ultrasound images demonstrating visible changes in tissue echogenicity for Patient 2 before (**A**) and after (**B**) therapy.

**Figure 6:**
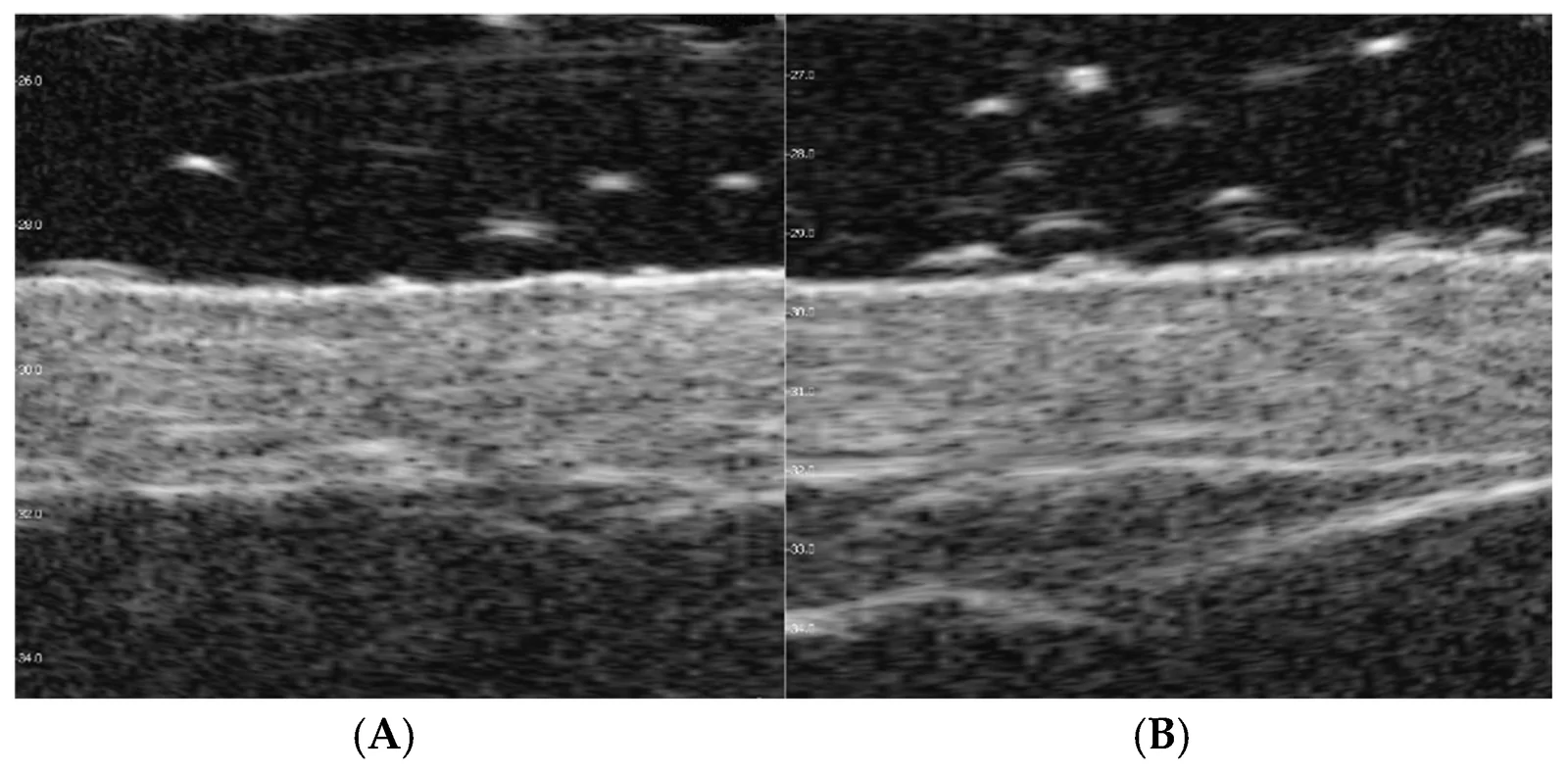
Ultrasound images demonstrating visible changes in tissue echogenicity for Patient 9 before (**A**) and after (**B**) therapy.

To evaluate structural and aesthetic improvements in striae distensae, pre- and post-treatment ultrasound images (Figure 5 and Figure 6) demonstrate changes in echogenicity that directly correspond with the quantitative data in Table 2 and Table 3.

Following the treatment, the majority of the study group (seven patients: P1–P3, P6–P9) demonstrated an increase in medium echogenicity alongside a decrease in high echogenicity. In these cases, ultrasound imaging revealed a more homogeneous arrangement of fibrous structures corresponding to collagen architecture, which perfectly aligns with the quantitative shifts in MEP/TP and HEP/TP parameters. Conversely, an atypical tissue response was observed in three patients (P4, P5, P10). Their ultrasound images displayed an opposing trend, characterized by increased overall echogenicity and a higher proportion of bright areas, corroborating their inverse quantitative results of decreased MEP/TP and increased HEP/TP values. Ultrasound images of patients P1, P3-P8 and P10 are presented in Supplementary Materials (Figures S1–S8).

Although visible qualitative improvements and structural tissue remodeling were observed via ultrasound imaging across the cohort, the quantitative parameters (such as MEP and TP) did not reach statistical significance (*p* > 0.05) due to the small sample size of this pilot study (*N* = 10). Therefore, these changes should be interpreted as observational trends reflecting consistent individual-level responses rather than definitive group-level statistical differences.

**Figure 7:**
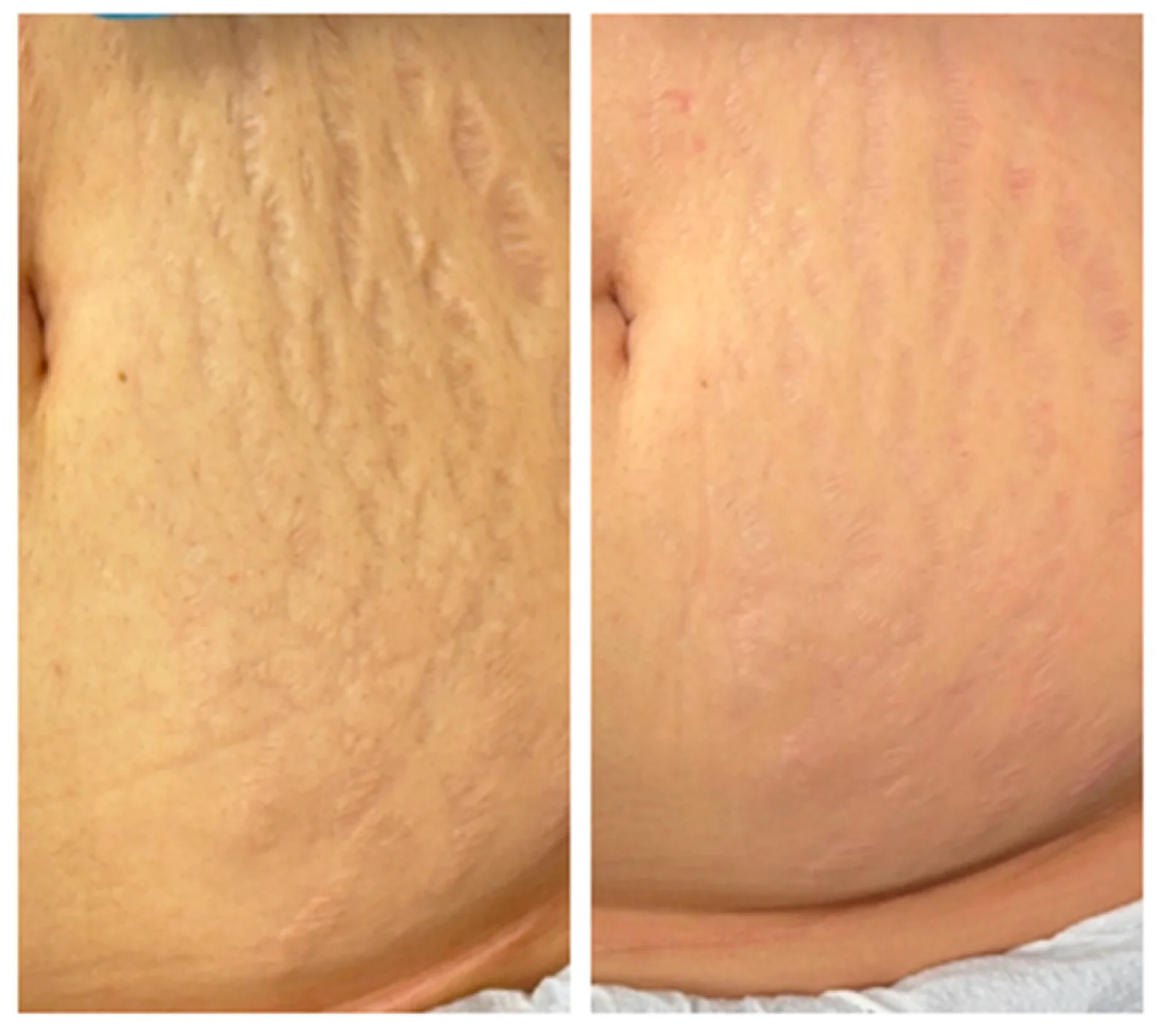
Visible changes before (**left**) and after (**right**) SD therapy of patient P2.

**Figure 8:**
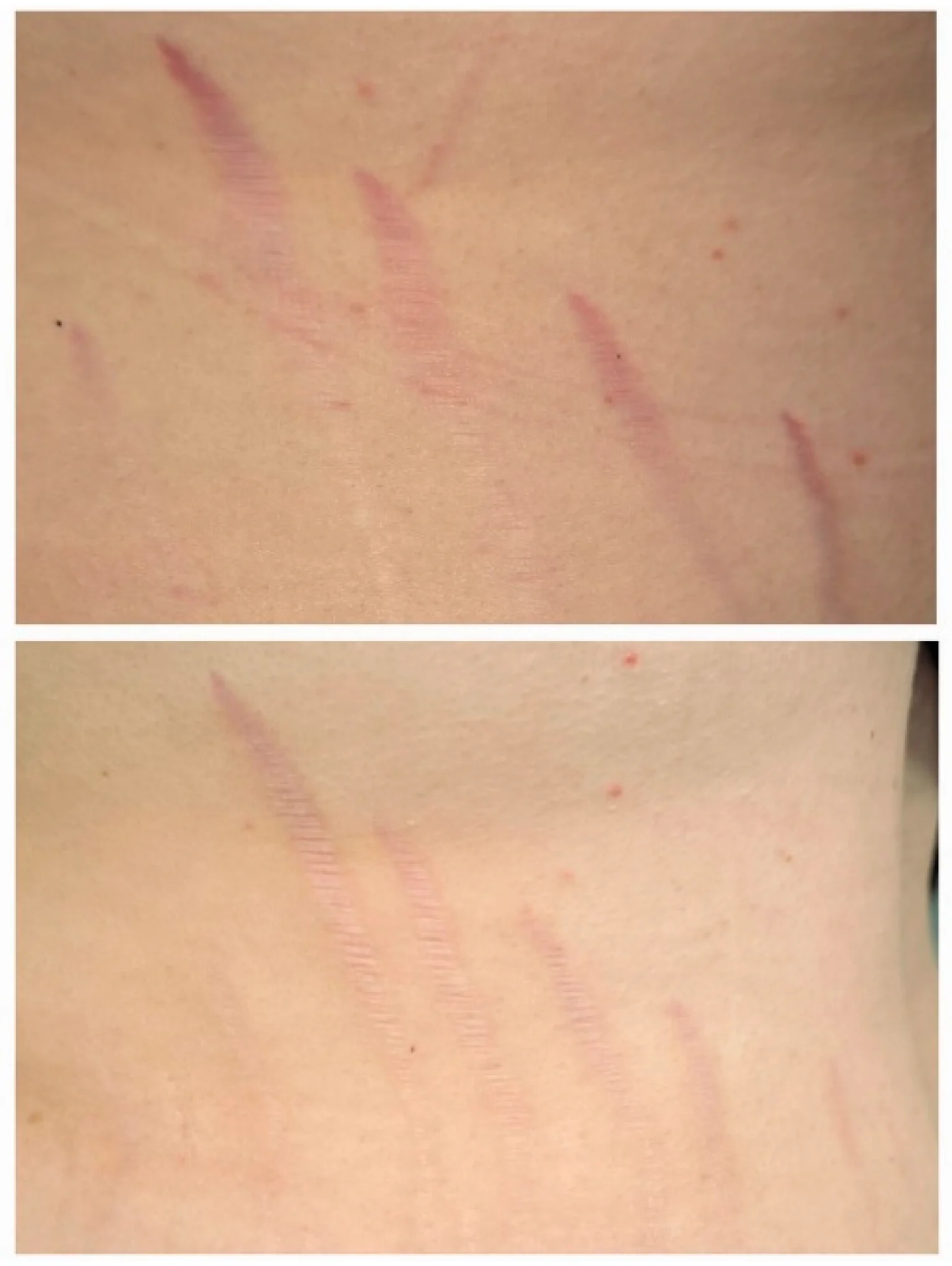
Visible changes before (**upper**) and after (**lower**) SD therapy of patient P4.

Finally, selected clinical photographs of the treatment area (Figure 7 and Figure 8) indicate that the deep structural tissue remodeling observed in the ultrasound translates into visible aesthetic improvements, manifested as a reduction in the depth and prominence of the striae distensae. The photos of patients P1, P7 and P10 are presented in Supplementary Materials (Figures S9–S11).

## 4. Discussion

Striae distensae are atrophic scars marked by collagen degradation, elastolysis, and extracellular matrix disruption [21,40]. Characterized by collagen degradation, elastolysis, and architectural disorganization [9], their evolution from rubrae to albae culminates in permanent structural damage and a loss of tissue elasticity [1,5,21]. Consequently, these lesions are not merely aesthetic defects, but reflect profound extracellular matrix disorders [41].

High-frequency ultrasound (>20 MHz) offers safe, real-time, high-resolution imaging for the objective evaluation of skin layer thickness, echogenicity, and architecture, making it an ideal tool for assessing connective tissue remodeling and striae severity [32,38,42].

This study demonstrated objective, measurable changes in skin structure following a series of microneedling treatments. Quantitative analysis of the USG images revealed an overall increase in the mean MEP/TP parameter and a decrease in the mean HEP/TP parameter in the study group, combined with a more homogeneous arrangement of fibrous structures observed in the ultrasound examination. In the vast majority of patients, a higher proportion of MEPs and a simultaneous decrease in HEP pixels were observed post-treatment. Such a distribution of parameters in the ultrasonographic image indicates the formation of a more homogeneous and organized arrangement of fibrous structures, corresponding to a new collagen architecture. These results are consistent with the pathomechanism of microneedling described in the literature. As demonstrated by Alster and Graham [43] and Singh and Yadav [44] the mechanical breakdown of old, dense collagen bundles stimulates the wound healing cascade and the process of neocollagenesis-a biological process stimulating fibroblasts to produce new collagen fibers. The scaffold formed during the remodeling phase, initially composed of type III collagen, transforms into stronger type I collagen. Therefore, the increase in medium echogenicity pixels (MEPs) confirms the gradual restoration of normal tissue density in the site of the previously disorganized matrix.

Similar conclusions can be drawn from analyzing the literature on skin ultrasound imaging. Comparable correlations were demonstrated by Bleve et al. [35], where the authors investigated methods for evaluating the efficacy of striae treatment. It was observed that pre-treatment striae are characterized by significant thinning of the dermis and a decrease in its echogenicity (an increased number of dark, anechoic areas), which clearly indicates a reduction in structural fibers and flattening of the dermo-epidermal junction. Similar studies conducted by Veronese et al. [10] proved that these are areas of significantly reduced structural cohesiveness. The shift in echogenicity towards medium MEP values, achieved in this study, provides measurable evidence of the reversal of atrophic processes and the densification of the skin’s collagen network.

The ultrasonographic observations were directly confirmed by the analysis of photographic documentation, which proved a clear clinical improvement in the patients. A reduction in the depth of the lesions, blurred and less sharp margins of the striae, a significant decrease in atrophy and fading of color were noted. The results of this study are highly consistent with reports by other authors, such as Abd El-Razek et al. [45] and Park et al. [14] who proved that microneedling is an effective, safe, and minimally invasive therapeutic method for reducing striae, noticeably improving their texture and pigmentation, and decreasing their overall length and width. Clinical studies indicate progressive aesthetic improvement for both early red striae and mature white striae, although lesions at an earlier stage generally exhibit a better response to treatment. This is explained by increased vascular activity and a greater potential for collagen remodeling during the active inflammatory phase [44]. A noticeable clinical improvement exceeding 50% is typically observed by Alster and Li [46] in patients after just one to three treatments performed at monthly intervals, and the tissue remodeling process and reduction in lesion visibility continue for 3 to 6 months after the treatment concludes. 

A significant advantage of microneedling, which drives its growing popularity, is its high safety profile, associated with the absence of thermal tissue damage, which is sometimes observed with laser therapies. This characteristic significantly reduces the risk of post-inflammatory hyperpigmentation (PIH), making this method a suitable option for patients with darker skin phototypes [1,7]. While non-ablative fractional lasers rely on thermal energy delivery—which carries a documented risk of post-inflammatory hyperpigmentation (PIH), particularly in darker skin types, needle-based mechanical stimulation induces collagen remodeling without thermal trauma [47]. Treatment efficacy remains at a similar level regardless of the body location of the lesions and the thickness of the skin itself. Satisfactory results are achieved both in areas with thicker tissue, such as the buttocks or thighs, and in regions with thinner skin, for example, on the breasts [7,43,46]. Side effects of the procedure are generally mild and include only transient erythema, mild edema, pinpoint bleeding, pain, and pruritus, which resolve spontaneously within a few days, resulting in a short recovery period [48,49].

A key parameter determining the efficacy of the therapy is the precisely selected depth of needle penetration. Because striae affect the deep dermis, the literature recommends 1.5 to 3.0 mm needles to effectively initiate structural repair [15]. Deeper punctures, utilizing needles of 1.5 mm or longer, extend the skin’s regenerative processes, translating into the necessity of maintaining at least a three-week interval between individual treatments [44,50]. However, it should be noted that working at a depth equal to or exceeding 3 mm is associated with significantly greater pain, which generally requires the prior application of appropriate local anesthesia [16].

It is important to note, however, that in three patients (P4, P5, P10), an inverse direction of changes in the USG image of the striae was recorded upon study completion. In these subjects, a decrease in the MEP/TP value and a simultaneous increase in the HEP/TP parameter (bright pixels) were observed-visible as a densification of bright areas on the ultrasound screen, which in some patients was extremely drastic. As stated by Veronese et al. [10] images with very high echogenicity (bright) are characteristic of surgical scars, which exhibit high stiffness, unlike flaccid striae. The distinct tissue response in these three patients may indicate a highly individualized, stronger fibroblastic response to the targeted injury and a more intense formation of dense, hyperplastic scar-like tissue at the site of the microwounds. The rapid increase in the HEP parameter may reflect an ongoing intense phase of proliferation and accumulation of newly synthesized collagen. Notably, among the three patients (P4, P5, and P10) who exhibited an inverse trend, patients P4 and P5 specifically presented with active striae rubrae at baseline (Table 1). This distinction is critical, as the active inflammatory phase and high vascularity inherent to striae rubrae—unlike the chronic, atrophic state of striae albae—significantly alter baseline tissue echogenicity and healing dynamics. Rather than unmeasured lesion dimensions, this underlying difference in lesion stage and microvascular architecture serves as the most plausible biological confounder driving the distinct quantitative response observed during the remodeling phase.

These observations suggest that despite the high safety profile of microneedling and short recovery time (as confirmed by Alster and Li [46] and Sanad et al. [48], the body’s response during the remodeling phase exhibits varied dynamics depending on the patient’s individual predispositions. As authors analyzing tissue remodeling processes note, the final formation of durable type I collagen and the creation of a functional elastic network can progress for up to 3 to 6 months after treatment completion. The differences in results between individual patients may stem from the exact moment of the remodeling phase at which the ultrasound examination was performed [15,18,43]. It is worth noting that the lack of conclusive results may have been influenced by the small sample size (*N* = 10) and high individual variability in response to therapy, as illustrated by extreme values in individual patients (e.g., patient P4 showed a decrease in the MEP/TP parameter by nearly 62%, accompanied by an unusually high, group-atypical increase in the HEP/TP parameter).

Combining microneedling with trichloroacetic acid (TCA) or platelet-rich plasma (PRP) significantly enhances efficacy compared to monotherapy [50]. Notably, pairing microneedling with 15–30% TCA increases collagen content and epidermal thickness [48], highlighting a promising direction for future research.

This study has certain limitations that must be considered when interpreting the results. Key limitations of this study include a small sample size—which limits generalizability and increases susceptibility to outliers in densitometric readings—and an exclusively female cohort, which precludes gender-based comparisons of striae architecture and treatment response. Despite strict procedural controls such as a single experienced operator and standardized device settings, this pilot study is limited by manual probe operation and the lack of automated stereotactic positioning. Due to the exploratory pilot nature of this study (*n* = 10), single ROI sampling, and the lack of blinded rating scales, our findings are interpreted strictly as observational trends rather than definitive group-level statistical conclusions, establishing a vital baseline for future blinded, multi-center trials.

## 5. Conclusions

High-frequency ultrasound (48 MHz) serves as a valuable non-invasive tool for monitoring structural skin changes; in this pilot sample, microneedling demonstrated a favorable safety profile and promising observational trends in tissue remodeling, supporting its consideration as a minimally invasive modality for striae distensae. Larger prospective studies are warranted to confirm broader quantitative statistical significance. Future research should utilize larger, mixed-gender cohorts and explore combination protocols to optimize clinical outcomes.

## Figures and Tables

**Table 1:** Characteristics of participant’s stretch marks.

Patient Number	Stage of Stretch Marks	Cause of Stretch Marks	Location	Previous Treatment
P1	striae albae	pregnancy	Abdominal	None
P2	striae albae	pregnancy		
P3	striae albae	pregnancy		
P4	striae rubrae	Rapid body weight increase		
P5	striae rubrae	Rapid body weight increase		
P6	striae albae	pregnancy		
P7	striae albae	pregnancy		
P8	striae albae	pregnancy		
P9	striae albae	pregnancy		
P10	striae albae	pregnancy		

**Table 2:** Descriptive statistics for the clinical parameters of the study group before and after treatment.

Parameter	Treatment Phase	Median	Q1–Q3
MEP/TP	Before	72.52	53.54–90.34
	After	81.25	73.41–86.18
HEP/TP	Before	28.47	7.84–47.70
	After	19.55	14.66–27.98

Q1-quartile first, Q3-quartile third.

**Table 3:** Individual patient results and percentage changes for MEP/TP and HEP/TP parameters before and after treatment.

Patient	MEP		MEP % of Change	HEP		HEP % of Change
	Before	After		Before	After	
P1	53.54	80.21	49.81%	47.70	20.47	−57.09%
P2	42.44	73.41	72.97%	58.82	27.98	−52.43%
P3	90.34	92.46	2.35%	7.84	0.93	−88.14%
P4 †	95.75	36.46	−61.92%	4.60	64.90	1310.87%
P5 †	93.68	91.51	−2.32%	5.94	8.96	50.84%
P6	70.20	82.28	17.21%	30.86	18.62	−39.66%
P7	68.15	76.14	11.72%	32.19	24.84	−22.83%
P8	39.76	86.18	116.75%	61.52	14.66	−76.17%
P9	77.14	83.36	8.06%	24.06	17.91	−25.56%
P10 †	74.84	70.41	−5.92%	26.08	30.62	17.41%

† Denotes patients (P4, P5, P10) whose clinical parameters trended in the opposite direction (decrease in MEP/TP, increase in HEP/TP) relative to the predominant trend observed in the rest of the group. Note: Minor deviations in combined percentage sums (e.g., 101.3%) in individual scans reflect independent automated rounding of pixel density categories within the native ultrasound analysis software.

## Data Availability

The original contributions presented in the study are included in the article. Further inquiries can be directed to the corresponding author.

## Supplementary Materials

The following supporting information can be downloaded at https://www.mdpi.com/article/10.3390/diagnostics16213444/s1: Figure S1. Ultrasound images demonstrating visible changes in tissue echogenicity for Patient 1 before (A) and after (B) therapy; Figure S2. Ultrasound images demonstrating visible changes in tissue echogenicity for Patient 3 before (A) and after (B) therapy; Figure S3. Ultrasound images demonstrating visible changes in tissue echogenicity for Patient 4 before (A) and after (B) therapy; Figure S4. Ultrasound images demonstrating visible changes in tissue echogenicity for Patient 5 before (A) and after (B) therapy; Figure S5. Ultrasound images demonstrating visible changes in tissue echogenicity for Patient 6 before (A) and after (B) therapy; Figure S6. Ultrasound images demonstrating visible changes in tissue echogenicity for Patient 7 before (A) and after (B) therapy; Figure S7. Ultrasound images demonstrating visible changes in tissue echogenicity for Patient 8 before (A) and after (B) therapy; Figure S8. Ultrasound images demonstrating visible changes in tissue echogenicity for Patient 10 before (A) and after (B) therapy; Figure S9. Visible changes before (upper photo) and after (lower photo) SD therapy of patient P1.; Figure S10. Visible changes before (upper photo) and after (lower photo) SD therapy of patient P7.; Figure S11. Visible changes before (left photo) and after (right photo) SD therapy of patient P10.
