# Supplementary material for: Application of High-Frequency Ultrasound (48 MHz) in Imaging and Evaluating the Effectiveness of Microneedling in Reducing Striae Distensae—A Pilot Study
Source: Diagnostics (Basel). 2026 Oct 22;16(21):3444. doi: 10.3390/diagnostics16213444 (PMC13761512; doi:10.3390/diagnostics16213444)
Supplement: Supplementary file 1 [file diagnostics-16-03444-s001.zip › diagnostics-4561791-supplementary.pdf]

Suplementarny material

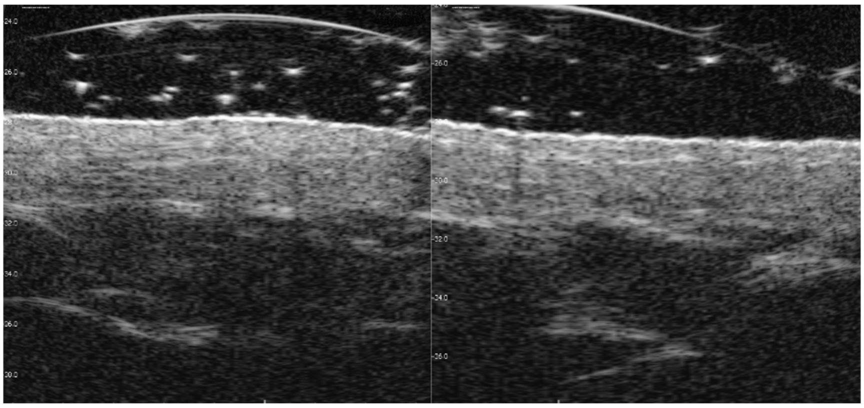

Figure S1. Ultrasound images demonstrating visible changes in tissue echogenicity for Patient 1 before (A) and after (B) therapy.

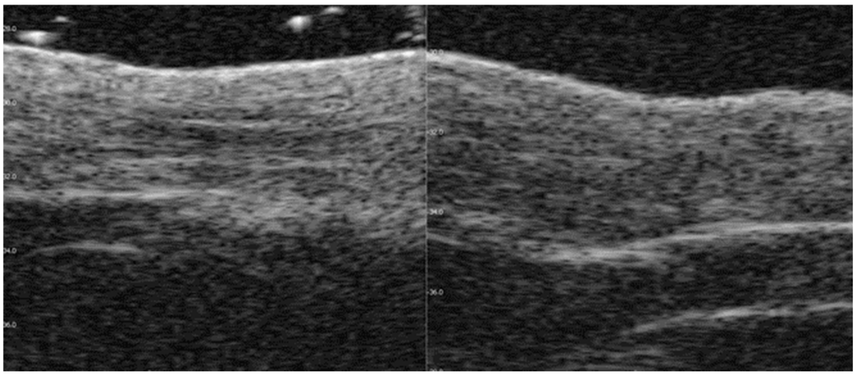

Figure S2. Ultrasound images demonstrating visible changes in tissue echogenicity for Patient 3 before (A) and after (B) therapy.

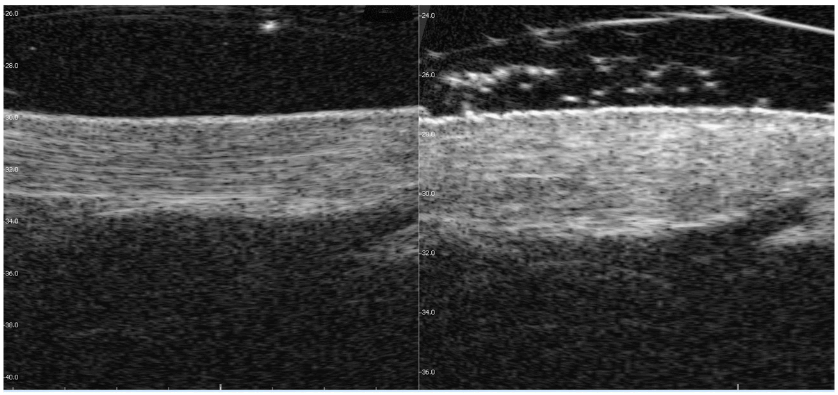

Figure S3. Ultrasound images demonstrating visible changes in tissue echogenicity for Patient 4 before (A) and after (B) therapy.

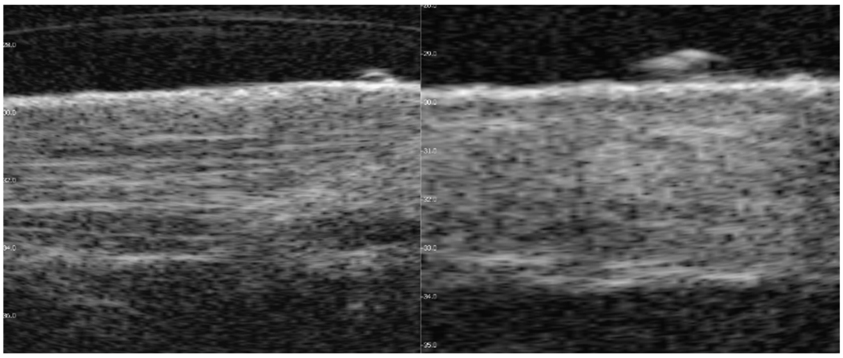

Figure S4. Ultrasound images demonstrating visible changes in tissue echogenicity for Patient 5 before (A) and after (B) therapy.

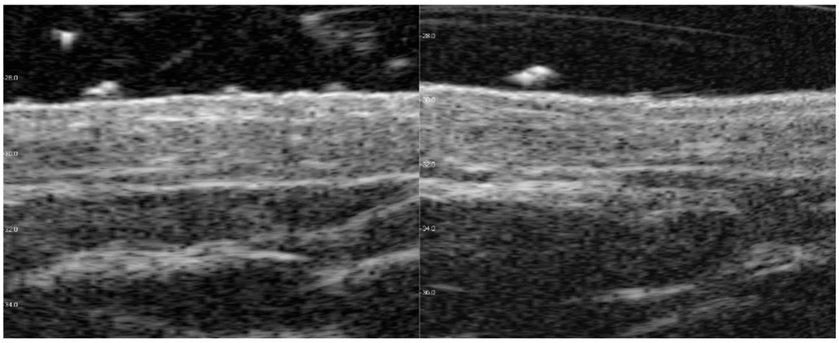

Figure S5. Ultrasound images demonstrating visible changes in tissue echogenicity for Patient 6 before (A) and after (B) therapy.

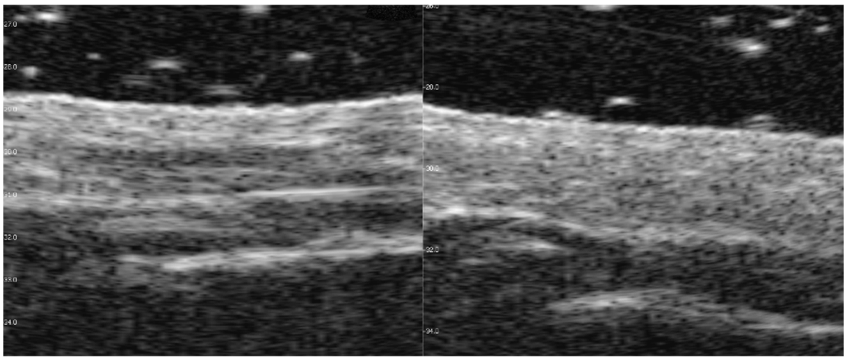

Figure S6. Ultrasound images demonstrating visible changes in tissue echogenicity for Patient 7 before (A) and after (B) therapy.

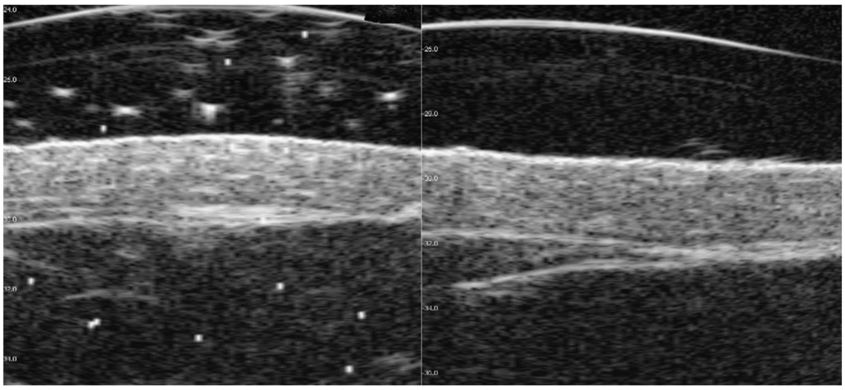

Figure S7. Ultrasound images demonstrating visible changes in tissue echogenicity for Patient 8 before (A) and after (B) therapy.

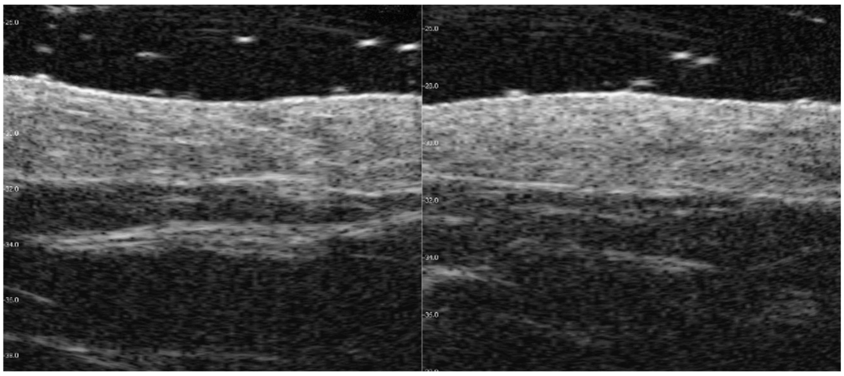

Figure S8. Ultrasound images demonstrating visible changes in tissue echogenicity for Patient 10 before (A) and after (B) therapy.

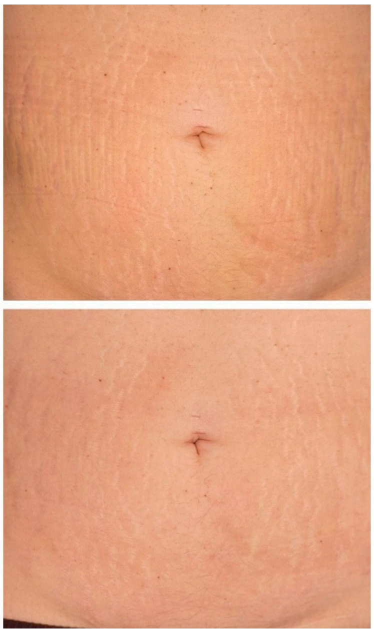

Figure S9. Visible changes before (upper photo) and after (lower photo) SD therapy of patient P1.

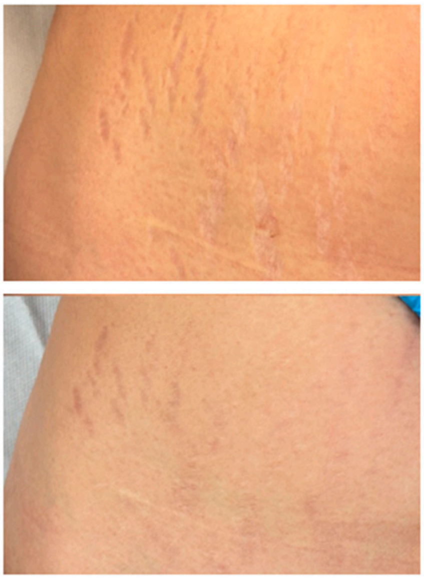

Figure S10. Visible changes before (upper photo) and after (lower photo) SD therapy of patient P7.

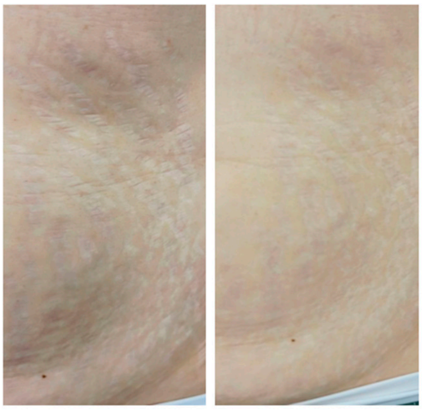

Figure S11. Visible changes before (left photo) and after (right photo) SD therapy of patient P10.
